# The Use of Blue Light as an Antimicrobial Treatment Against *Staphylococcus aureus*: A Systematic Review

**DOI:** 10.1002/lsm.70172

**Published:** 2026-07-15

**Authors:** Alexander Idrogo‐Lam, Kofi Owusu‐Ansah, Eric Cotter, Lisa Cotter

**Affiliations:** ^1^ University of Wisconsin School of Medicine and Public Health Madison Wisconsin USA; ^2^ Department of Orthopedics and Rehabilitation University of Wisconsin School of Medicine and Public Health Madison Wisconsin USA; ^3^ Department of Dermatology University of Wisconsin School of Medicine and Public Health Madison Wisconsin USA

## Abstract

**Background:**

*Staphylococcus aureus (S. aureus)* is a major cause of skin, wound, and device‐related infections. Blue light (405–470 nm), shows promise for treating this organism. However, the translatability, dosing parameters, safety, and effectiveness remain unclear, and no systematic review currently exists.

**Objectives:**

To evaluate the antimicrobial efficacy and safety of blue light therapy against *S. aureus* across experimental models.

**Methods:**

All relevant in vitro, in vivo, and clinical studies were identified through systematic searches of PubMed, Embase, Web of Science, Cochrane Library, Scopus, and ClinicalTrials. gov. Studies evaluating blue light without exogenous photosensitizers reporting bacterial or clinical outcomes were included. Risk of bias was assessed using SYRCLE. This study was registered with PROSPERO (ID: CRD420251089518).

**Results:**

A total of 33 articles met inclusion criteria: 20 planktonic models, 6 biofilm models, and 17 methicillin‐resistant *S. aureus* (MRSA) specific outcomes. The predominant wavelength tested was 405 nm (87.9%). Blue light reduced bacterial burden across models, though results varied by dose and experimental conditions. Additionally, 57.6% of studies did not report statistical significance.

**Conclusions:**

Blue light demonstrates antimicrobial activity against *S. aureus*, including MRSA, but heterogeneity in study design and limited in vivo data restrict clinical translation. We propose a standardized set of 8 reported parameters for future blue light studies.

## 1 Introduction

Blue light (BL), spanning 405–470 nm, has garnered attention in dermatology and orthopedics as an antimicrobial treatment [1, 2, 3]. This technology exerts its biocidal effects by photoexciting endogenous microbial chromophores, expressed to a greater extent in bacteria, leading to generation of reactive oxygen species (ROS). ROS, in turn, causes oxidative cellular damage and ultimately the death of the microorganisms [4].

The use of BL as an antimicrobial treatment against *Staphylococcus aureus (S. aureus)* including methicillin‐resistant *S. aureus* (MRSA) is an important and emerging area of research, particularly due to its non‐antibiotic mechanism. These bacteria are often responsible for a substantial portion of skin infections, wound infections, and the formation of biofilms on medical devices. Previous research has demonstrated promising results in both laboratory and animal studies, showing that BL effectively reduces the viability of *S. aureus* [5, 6, 7, 8, 9, 10, 11, 12]. However, the translatability, dosing parameters, safety, and effectiveness of Blue Light Therapy (BLT) for *S. aureus* remain unclear.

To date, no comprehensive work has investigated treatment effect of BLT against *S. aureus* without the use of exogenous photosensitizers. Clinically, the use of photosensitizers is not optimal, as they can induce keratinocyte apoptosis, pain, erythema, and skin barrier breakdown [13, 14]. Therefore, this systematic review aims to aggregate existing data pertaining to the utilization of BLT, without the use of exogenous photosensitizers, as an antimicrobial treatment against *S. aureus*, identifying areas that warrant further investigation.

## 2 Materials and Methods

### 2.1 Literature Search

A comprehensive literature search was conducted in PubMed/MEDLINE, Embase, Web of Science, the Cochrane Library, Scopus, and ClinicalTrials.gov from database inception through June 2025. This systematic review was registered in the PROSPERO database (ID: CRD420251089518). Search strings were constructed using a combination of Medical Subject Headings (MeSH) and free‐text terms encompassing “*Staphylococcus aureus*,” “MRSA,” “blue light,” and “phototherapy,” with related synonyms adapted for each database. The search strategy adhered to no specific date restrictions and exclusively encompassed English‐language, peer‐reviewed publications. Two independent reviewers (A.I.L. and K.O.A.) conducted comprehensive title, abstract, and full‐text screenings. The risk of bias for in vivo studies was assessed using the SYRCLE Risk of Bias tool [15]. For ex vivo and in vitro studies, key methodological parameters relevant to reproducibility and dose delivery were reviewed, given the absence of validated Risk of Bias tools for these designs.

### 2.2 Eligibility Criteria

Studies were eligible for inclusion if they specifically investigated the effectiveness of BL exposure at wavelengths ranging from 405 to 470 nm. Combined approaches were allowed if the effects of BL exposure alone could be distinguished. Additionally, included studies must have been focused on *S. aureus*, including MRSA. The study types included in our review were in vitro, in vivo, and clinical studies. Furthermore, each study must have reported a bacterial load, colony‐forming units (CFUs), or clinical infection resolution. Studies excluded were those that did not specifically report outcomes on *S. aureus*, explored photodynamic therapy with chemical photosensitizers, or did not separate the effects of BL exposure alone. Reviews, editorials, and conference abstracts were also excluded unless sufficient data was available. This systematic review followed the Preferred Reporting Items for Systematic Reviews and Meta‐analyses 2020 reporting guideline statement [16].

### 2.3 Data Extraction

For all included publications, two reviewers (A.I.L. and K.O.A.) independently screened the title and abstracts of included trials. Publications meeting screening criteria then underwent full text reviews. A third author (L.C.) facilitated a consensus discussion to resolve any disputes regarding the inclusion of literature. Extracted variables from the study encompassed the study type, organism, model type, and irradiation parameters (wavelength, dose, exposure time). The primary outcome of interest was the reduction in CFU counts following BL treatment. When CFU data was not reported, the overall trends in infection resolution or bacterial inhibition were assessed. The magnitude and statistical significance of the observed effects and the statistical methods used were also evaluated. Secondary outcomes encompassed the incidence of adverse effects, including cytotoxicity or other reported tissue responses. Statistical analysis was limited to descriptive interpretation, and no comparative or meta‐analysis was performed due to the heterogeneity of the included studies.

## 3 Results

A total of 4252 articles were extracted from the data, and 2105 articles were screened after removing duplicates. Out of these, 45 studies met the criteria for a full‐text review, but three articles were unable to be retrieved. Consequently, 33 studies met the criteria for inclusion, as seven were excluded due to the use of photosensitizers, and two were excluded due to incorrect BL wavelength (Figure S1).

A total of 33 publications met full eligibility criteria (Table S1). Notably, 17 publications (51.5%) reported MRSA specific outcomes. The most frequently employed wavelength was 405 nm, tested in 87.9% of the publications. Delivered doses varied significantly across studies, with the lowest dose being 1.08 J/cm^2^ and the highest being 3240 J/cm^2^. Thirty‐one publications utilized in vitro models (93.9%), with only four publications using in vivo models (12.1%). Publications with ex vivo experiments were among the least studied, with only one being examined (3.0%).

### 3.1 Planktonic Versus Biofilm Models

Across 20 planktonic *S. aureus* models (Table 1), BLT demonstrated consistent bactericidal activity. Reductions in bacterial burden were dose dependent, with 3–6 log_10_ CFU reductions observed across dosages of 36–720 J/cm^2^. Nevertheless, five experiments reported nonsignificant results (25%) and nine did not comment on statistical significance (45%). Both MRSA and MSSA strains exhibited overlapping susceptibility profiles, with no consistent differences in response attributable to methicillin resistance status.

**Table 1:** Planktonic studies (*n* = 16). In certain publications, multiple time points were evaluated. However, data was excluded from studies that did not provide results for the specified time points. Consequently, no time points were included in the table. If a time point was included but no results were specified, it was not included in the table. Outcome summaries labeled with a (*) indicates that the statistical significance was not specified. Outcome summaries denoted with (**) indicated that the time point was not statistically significant. Tables were organized by dose, with the highest dose listed first and subsequent rows displaying progressively lower doses.

Study identification	Model type	Organism/Strain	Wavelength (nm)	Dose (J/cm^2^)	Exposure time	Outcome summary
Dos Anjos et al. [17]	PBS/Milk	MSSA	413	720	120 min	> 5 log_10_ reduction
Dos Anjos et al. [18]	Bacterial suspension	MSSA	410 ± 10	63.5–549.6	Not reported	1.0–4.54 log_10_ reduction*
Al‐Shammary et al. [19]	Liquid culture	MRSA	405	480	10 min	6% survival*
Hoenes et al. [20]	Bacterial suspension	MRSA	405;450	160–400	Not reported	1.22–5.69 log_10_ reduction*
Tomb et al. [21]	Bacterial suspension	MRSA	405	324	90 min	5.3–5.4 log_10_ reduction (15 cycles)**
Maclean et al. [22]	Platelet concentrates	MSSA	405	64.8–288	5–8 h	> 92.5%–99.6% reduction
Rapacka‐Zdonczyk et al. [23]	Planktonic	MSSA	411	50–220	16–20 h	2–3 log_10_ reduction*
Yang et al. [24]	Planktonic	MSSA	460	30–270	10–90 min	0.64–4.11 log_10_ reduction
Tomb et al. [21]	Bacterial suspension	MSSA	405	270	75 min	4.8–5.2 log_10_ reduction (15 cycles)**
Biener et al. [25]	Bacterial culture	MRSA	405	121–242	15–30 min	~3%–10% survival
Mu et al. [26]	Planktonic	MSSA	453	3.45–165.6	2.5–120 min	Small decrease to decrease**
Gillespie et al. [27]	Liquid suspension	MSSA	405	17.4–115	30–120 min	48.9%–99.8% reduction
Tomb et al. [21]	Bacterial suspension	MSSA	405	108	30 min	1.2–1.3 log_10_ reduction (15 cycles)**
Tomb et al. [21]	Bacterial suspension	MRSA	405	108	30 min	1.3–1.4 log_10_ reduction (15 cycles)**
Wu et al. [5]	Bacterial suspension	MRSA	415	80	Not reported	> 6 log_10_ reduction*
Zhang et al. [28]	Planktonic	MRSA	405	36	40 min	5 log_10_ reduction
Al‐Shammary et al. [19]	Liquid culture	MRSA	405	Not reported	1 min	60% survival*
Al‐Shammary et al. [19]	Liquid culture	MRSA	405	Not reported	2 min	30% survival*
Al‐Shammary et al. [19]	Liquid culture	MRSA	405	Not reported	5 min	6% survival*
Ong et al. [29]	Planktonic bacteria	MSSA	405	Not reported	2.5 h	3.0 log_10_ reduction*

Abbreviations: MRSA, methicillin‐resistant *Staphylococcus aureus*; MSSA, methicillin‐susceptible *Staphylococcus aureus*.

When comparing to planktonic cultures, biofilm associated *S. aureus* demonstrated reduced susceptibility to BLT (Table 2). There were six studies that evaluated biofilm models, which consisted of 13 different experiments, including static and dynamic biofilms. Reported effect sizes were generally smaller than those observed in planktonic systems, with reductions ranging from 1 to 3.48 log_10_ CFU reductions. Of these experiments, five reported nonsignificant antimicrobial effects (38.5%).

**Table 2:** Biofilm studies (*n* = 6). In certain publications, multiple time points were evaluated. However, data was excluded from studies that did not provide results for the specified time points. Consequently, no time points were included in the table. If a time point was included but no results were specified, it was not included in the table. Outcome summaries labeled with a (*) indicates that the statistical significance was not specified. Outcome summaries denoted with (**) indicated that the time point was not statistically significant. Tables were organized by dose, with the highest dose listed first and subsequent rows displaying progressively lower doses.

Study identification	Model type	Organism/Strain	Wavelength (nm)	Dose (J/cm^2^)	Exposure time	Outcome summary
Halstead et al. [30]	Biofilm	MSSA	405–450	407.2–513	19.5–45 min	Variable biofilm effects**
Espada et al. [31]	Static biofilm (24 h)	MRSA	405	500	90 min	3.48 ± 0.39 log_10_ reduction
Espada et al. [31]	Static biofilm (48 h)	MRSA	405	500	90 min	2.35 ± 0.23 log_10_ reduction
Espada et al. [31]	Dynamic biofilm	MRSA	405	500	90 min	2.58 ± 0.30 log_10_ reduction**
Espada et al. [32]	MRSA biofilm	MRSA	405	216	60 min	24 h biofilm: 1.58 log_10_ reduction; 48 h biofilm: 0.20 log_10_ reduction*
Espada et al. [31]	Static biofilm (24 h)	MRSA	405	216	60 min	1.2 ± 0.20 log_10_ reduction
Espada et al. [31]	Static biofilm (48 h)	MRSA	405	216	60 min	1.62 ± 0.13 log_10_ reduction
Espada et al. [31]	Dynamic biofilm	MRSA	405	216	60 min	2.05 ± 0.33 log_10_ reduction**
Bumah et al. [33]	Planktonic/biofilm	MRSA	450	4.5–10.8	1500–5400 s	0.8–7.0 log_10_ reduction
Rutala et al. [34]	Formica sheet	MRSA	405 ± 3	Not reported	0–72 h	25%–90% reduction over time
Ong et al. [35]	Planktonic/biofilm	MSSA	405	Not reported	2.5–10 h	0–3.0 log_10_ reduction*
Ong et al. [29]	Sheep skin + Ti implants	MSSA	405	135; 270; 540	15–60 min	No statistical difference vs. pretreatment
Ong et al. [29]	Sheep skin + Ti implants	MSSA	405	1080; 3240	6 h/day; 1 h twice daily (3 days)	24 h: 1.63 log_10_ reduction; 72 h: ~2.7 log_10_ reduction

Abbreviations: MRSA, methicillin‐resistant *Staphylococcus aureus*; MSSA, methicillin‐susceptible *Staphylococcus aureus*.

One study reported the differences between static and dynamic biofilms, where dynamic biofilms, attempting to replicate in vivo environment, did not show a statistically significant reduction compared to static biofilms [31]. Biofilm maturity influenced responsiveness, with earlier‐stage biofilms demonstrating greater susceptibility than established or mature biofilms in this study [31]. Eleven of the 13 biofilm experiments required energy levels exceeding those typically considered clinically feasible (84.5%) as keratinocyte damage is reported around 100 J/cm^2^ [36, 37, 38].

Notably, one study directly evaluated both planktonic and biofilm conditions within the same experimental framework, demonstrating reduced susceptibility in biofilm‐associated bacteria relative to planktonic counterparts under the same BL parameters [29].

### 3.2 In Vivo and Ex Vivo Models

In vivo and ex vivo experiments were reported in five studies (Table 2). In vivo models primarily consisted of wound infection models in mammals.

All five in vivo and ex vivo studies demonstrated reductions in bacteria following BL exposure, with all but one reporting statistically significant decreases in CFU counts compared with untreated controls (20%). Some investigations noted side effects including: skin desiccation mitigated by hydration, thermal damage without cooling and skin necrosis at 20–30 mW/cm^2^ [29, 39, 40]. However some studies noted no visible apoptosis of mouse skin or thermal damage when BLT was coupled with a cooler [40, 41]. Risk of bias assessment was performed for in vivo animal models using the SYRCLE risk of bias tool. Most studies showed unclear risk of bias due to limited reporting of randomization, allocation concealment, and blinding. However, one study explicitly reported a lack of investigator blinding, resulting in a high overall risk of bias [39]. Selective outcome reporting and variability in irradiation parameters were common sources of potential bias.

### 3.3 MRSA‐Specific Outcomes

MRSA‐specific experiments comprised a substantial proportion of included studies (Table 3). BLT demonstrated antimicrobial activity against multiple MRSA strains across planktonic, biofilm, and in vivo models. Reported CFU reductions for MRSA were comparable to those observed for MSSA under similar irradiation parameters. However, none of the MRSA‐specific experiments report significance when compared to control.

**Table 3:** MRSA studies (*n* = 17). In certain publications, multiple time points were evaluated. However, data was excluded from studies that did not provide results for the specified time points. Consequently, no time points were included in the table. If a time point was included but no results were specified, it was not included in the table. Outcome summaries labeled with a (*) indicates that the statistical significance was not specified. Outcome summaries denoted with (**) indicated that the time point was not statistically significant. Tables were organized by dose, with the highest dose listed first and subsequent rows displaying progressively lower doses.

Study identification	Strains tested	Dose (J/cm^2^)/Irradiance (mW/cm^2^)	Exposure time	MRSA outcome
Zhang et al. [28]	MRSA	Not reported	10; 20; 30 min	86.63 ± 6.10%; 95.21 ± 2.01%; 99.96 ± 0.03% bactericidal rate *
Negri et al. [40]	MRSA USA300	250 J/cm^2^	24–96 h post‐infection	1.5–4.7 log_10_ reduction *
Rutala et al. [34]	MRSA ATCC 43300	0.34–0.44 mW/cm^2^	Up to 72 h	25%–90% reduction over time*
Espada et al. [32]	MRSA MEEI IB0004	216 J/cm^2^	60 min	24 h biofilm: 1.58 log_10_ reduction; 48 h biofilm: 0.20 log_10_ reduction*
Espada et al. [31]	MRSA USA300	216 J/cm^2^; 500 J/cm^2^	60–90 min	1.2–3.48 log_10_ reduction*
Tomb et al. [21]	MRSA EMRSA‐15	108–324 J/cm^2^	30–90 min	1.3–5.4 log_10_ reduction*
Al‐Shammary et al. [19]	MRSA ATCC 43300	200 mW/cm^2^	1–10 min	40%–94% survival*
Biener et al. [25]	MRSA USA300 ATCC BAA‐1680	121 J/cm^2^; 242 J/cm^2^	15–30 min	~3%–10% survival*
Wu et al. [5]	MRSA252	80 J/cm^2^	Not reported	> 6 log_10_ reduction*
Leanse et al. [41]	USA300 (MRSA)	108–324 J/cm^2^	15–105 min	0.64–3.58 log_10_ reduction*
Hoenes et al. [20]	*S. aureus* ATCC 43300 (MRSA)	90–315 J/cm^2^	Not reported	1.22–5.69 log_10_ reduction*
Hui et al. [39]	MRSA USA300	54–738 J/cm^2^	3–41 h	Growth inhibition‐2.0 log_10_ reduction*
Bumah et al. [42]	MRSA USA300	45–220 J/cm^2^	Not reported	> 3 log_10_ reduction*
Bumah et al. [33]	MRSA ATCC BAA‐1680	4.5–10.8 J/cm^2^	1500–5400 s	0.8–7.0 log_10_ reduction*
Bumah et al. [43]	MRSA USA300	108–262 J/cm^2^	60–144 min	Complete suppression*
Bumah et al. [44]	MRSA USA300	55 J/cm^2^	30.6 min	90.2%–97.3% suppression*
Masson‐Meyers et al. [45]	MRSA USA300	40–121 J/cm^2^	15–240 min	0%–10% survival*

Abbreviation: MRSA, methicillin‐resistant *Staphylococcus aureus*.

### 3.4 Dose Thresholds and Low‐Dose Exposure

There were 21 studies that evaluated BL with doses under 100 J/cm^2^ (Table 4). This is an important limiting factor with regards to translation as keratinocyte damage has been reported around 100 J/cm^2^ [36, 37, 38]. Studies within this range mostly demonstrated BL being effective for reducing planktonic bacteria but less effective for biofilms. Exposure times varied from 2.5 min to 6 h, with prolonged exposure time demonstrating greater bacterial reduction.

**Table 4:** Studies with blue light doses under 100 J/cm^2^ (*n* = 17). In certain publications, multiple time points were evaluated. However, data was excluded from studies that did not provide results for the specified time points. Consequently, no time points were included in the table. If a time point was included but no results were specified, it was not included in the table. Outcome summaries labeled with a (*) indicates that the statistical significance was not specified. Outcome summaries denoted with (**) indicated that the time point was not statistically significant. Tables were organized by dose, with the highest dose listed first and subsequent rows displaying progressively lower doses.

Study identification	Model type	Dose(s) < 100 J/cm^2^ tested	Exposure time	Outcome at < 100 J/cm^2^
Mu et al. [26]	Planktonic bacteria	3.45; 10.35; 17.25; 41.4; 82.8	2.5; 7.5; 12.5; 30; 60 min	Small decrease to 63.3% reduction
Nguyen et al. [46]	Agar plate	11.51; 34.52; 46.03; 57.54; 80.56	5; 15; 25; 35 min	15%–93% inactivation
Wu et al. [5]	Bacterial suspension in PBS	80	Not reported	> 6 log_10_ reduction
Shehatou et al. [47]	Liquid culture (MH medium)	62.7 ± 17.7	Not reported	10^2^ reduction
Shehatou et al. [47]	Liquid culture (human serum)	62.7	Not reported	1.65 log_10_ reduction
Rapacka‐Zdonczyk et al. [23]	Planktonic culture	50	Not reported	3.0 log_10_ reduction
Leanse et al. [41]	Planktonic bacteria	54	15 min	0.36 log_10_ reduction
Gillespie et al. [27]	Liquid auspension	17.4; 34.8; 43.2; 64.8	30; 40; 45 min	77% survival to 70.8% reduction
Sinclair et al. [48]	Surface‐seeded on agar	1.08; 1.8; 2.16; 3.6; 7.2	1; 2; 4; 6 h	15.2%–99.3% reduction
Hui et al. [39]	Rat abrasion wound	54	3 h	Signal decrease
Dos Anjos et al. [18]	Bacterial suspension	63.5	Not reported	1.0 log_10_ reduction
Maclean et al. [22]	Seeded platelet concentrates	64.8	6 h	> 92.5% reduction
Bumah et al. [42]	Bacterial culture on agar plate	45; 50; 55; 60	Not reported	> 3 log_10_ reduction
Bumah et al. [33]	Planktonic culture	4.5; 5.4; 7.6	1500; 1800; 2533 s	0.8; 3.0;7.0 log_10_ reductions
Bumah et al. [33]	Forming biofilm	7.6	3800 s	53% survival
Bumah et al. [44]	Bacterial culture on agar plate	55	30.6 min	90.2%–97.3% suppression
Masson‐Meyers et al. [45]	Bacteria in agar	40; 54	15 min (1x, 2x, 3x)	0.5%–10% survival
Yang et al. [24]	Planktonic cells	30; 90	10; 30 min	0.64 ± 0.07 log_10_ reductions

Abbreviation: PBS, phosphate‐buffered saline.

### 3.5 Statistical Reporting and Outcome Measures

Statistical reporting was inconsistent across included studies. 19 of 33 publications (57.6%) did not report statistical significance measures for antimicrobial outcomes, which limits interpretability and cross‐study comparison. Outcome reporting also varied substantially, with efficacy expressed as CFU log_10_ reductions, percent survival, percent inhibition, or even qualitative descriptors of growth suppression. Of included studies, five did not report the dose of BL tested (15.2%) (Table 5).

**Table 5:** Quality of evidence summary for included studies.

Study design category	Number of articles	Quality assessment method	Key quality finding
In vivo (animal)	4	SYRCLE risk of bias tool	Risk of bias was predominantly unclear; a detailed domain‑by‑domain assessment appears in Table 2.
Ex vivo (tissue)	1	No validated tool available	For ex vivo studies, key methodological parameters relevant to reproducibility and dose delivery were reviewed, given the absence of validated risk of bias tools for these designs.
In vitro only	28	No validated tool available	For in vitro studies, key methodological parameters relevant to reproducibility and dose delivery were reviewed, given the absence of validated risk of bias tools for these designs.
Clinical	0	N/A	No human clinical studies were identified.

## 4 Discussion

This systematic review summarizes current evidence evaluating BLT as a non‐antibiotic antimicrobial approach against *S. aureus*, including MRSA. Across 33 studies, BL exposure reduced bacterial burden in most experimental models, supporting its plausibility as an antimicrobial modality. However, substantial variation in study design, dosing parameters, and outcome reporting limits definitive conclusions regarding optimal treatment parameters or clinical applicability.

One key exclusion to this review was the omission of studies utilizing exogenous photosensitizers. This was done to isolate the antimicrobial effects of BL alone; although external chromophores may enhance ROS, clinical use of photosensitizers can induce keratinocyte apoptosis, pain, erythema, and skin breakdown [13, 14]. By focusing on BLT exposure alone, this review aimed to evaluate its intrinsic antimicrobial potential.

### 4.1 Planktonic Versus Biofilm *S. aureus*

A trend among included studies was the reduced susceptibility of biofilm‐associated bacteria compared with planktonic organisms. This observation aligns with established biofilm biology, in which extracellular polymeric matrices, altered metabolic activity, and reduced penetration of external stressors contribute to antimicrobial tolerance [49]. Biofilm‐associated infections represent a clinically relevant challenge, and the present findings suggest that BL monotherapy may not be ideal for eradication of biofilms at clinically acceptable doses. Nevertheless, BLT may still represent a viable strategy for decontamination of medical devices and implants, where keratinocyte safety is not a limiting concern.

### 4.2 Dose Considerations

One consideration noted in both in vivo and ex vivo studies was the balance between antimicrobial activity and tissue safety. While higher doses were generally associated with greater bacterial reductions, many studies demonstrating antimicrobial effects utilized doses that may approach ranges associated with keratinocyte injury or oxidative stress in experimental models [50, 51]. Importantly, antimicrobial effects were still observed in several studies at doses below 100 J/cm^2^, although results were less consistent, particularly in biofilm and in vivo systems. Future studies must prioritize evaluating antimicrobial activity and tissue safety to determine clinically relevant dosing parameters.

An additional limiting factor is that 5 of 33 studies did not report the dose of BL in the experiment, and no study mentioned the use of a light sensor to confirm the dose delivered in the experiment. Studies have shown that the dosing output of clinical phototherapy units can differ greatly from manufacturer specifications, with irradiance often measuring lower to even half the manufacturer specified dosing output [52, 53]. Without true dosing verification, light exposure in these clinical trials remains unclear, making dose–response comparisons between experiments and reproducibility nearly impossible.

Safety reporting was inconsistent across in vivo studies, and direct assessment of keratinocyte viability beyond visible inspection was rarely incorporated into study design. As a result, the existing evidence does not yet clearly establish whether doses capable of producing reliable reductions in bacterial burden can be delivered safely in human skin, particularly in the setting of repeated exposure. One included study investigated continuous low dose BLT (5 mW/cm^2^) as a safer alternative by maintaining bacteriostatic levels of BL on the skin, but exposure time (4–41 h) may be beyond what is clinically feasible [39]. On the other hand, one study found that sub‐lethal BL exposure may induce tolerance in *S. aureus* [23]. Further studies should be done to investigate if sub‐lethal exposure consistently promotes adaptive responses. The predominance of in vitro studies and limited in vivo data represents another significant gap. While in vitro models provide valuable mechanistic insights and dose‐response relationships, they cannot fully replicate the biological environment of a human infection.

### 4.3 Need for Standardized Reporting and Proposed Standardized Reporting Parameters

A major limitation identified across the literature was the lack of standardized reporting. Many studies reported wavelength without specifying dose. Since antimicrobial effects are dose dependent and influenced by delivery parameters, incomplete reporting limits reproducibility and prevents identification of optimal treatment protocols [12]. Similarly, variability in outcome measures prevented quantitative comparison or meta‐analysis. For this reason, to improve reproducibility and enable comparison, we propose a standardized set of reported parameters for future BLT studies:

1. Wavelength (nm): The specific peak emission wavelength of the light source used.
2. Dose (J/cm^2^): The total delivered exposure at the treatment surface.
3. Irradiance (mW/cm^2^): The power density at the treatment surface.
4. Exposure time: The total time required to deliver the dose.
5. Source‐to‐surface distance (cm): The distance between the light‐emitting surface and the target, as irradiance varies substantially with distance and is rarely standardized across studies.
6. Dose verification sensor: Whether an independent light sensor was used to confirm delivered dose, given documented discrepancies between manufacturer‐specified and actual device output [52, 53].
7. Bacterial outcome measure: CFU log_10_ reductions should be reported as the primary quantitative metric. Qualitative descriptors alone are insufficient for cross‐study comparison.
8. Clinically relevant tissue responses (for in vivo studies): Systematic documentation of any macroscopic or histological skin changes, including erythema, desiccation, or evidence of thermal injury, should be reported to facilitate safety assessment across studies.

### 4.4 Clinical Implications

BLT is already used clinically in dermatology, where antimicrobial effects against *Cutibacterium acnes* occur within dosing ranges tolerable to human skin, and is currently being investigated in orthopedics [1, 2, 3]. In contrast, application for *S. aureus* infections remains investigational. The findings of this systematic review suggest potential roles for BL in superficial infection control, wound decontamination, or adjunctive antimicrobial strategies, particularly where repeated noninvasive treatment is feasible. Combination approaches with conventional antimicrobials or other non‐antibiotic modalities may enhance efficacy while allowing lower BL doses.

BLT may also hold promise for patients with chronic inflammatory skin conditions associated with recurrent or persistent *S. aureus* colonization, such as epidermolysis bullosa [54]. In these populations, long‐term antibiotic use carries risks of antibiotic resistance and systemic side effects, making non‐antibiotic, topically applied antimicrobial modalities especially helpful. Additionally, while this review focused on *S. aureus*, the broader applicability of BLT to pseudomonas aeruginosa or other major causes of wound infections warrants ongoing investigation.

### 4.5 Limitations

This review is limited by the predominance of in vitro studies, limited in vivo data, and substantial heterogeneity in experimental design and reporting. Publication bias toward positive antimicrobial findings is possible. The lack of standardized safety assessment across studies limits definitive conclusions regarding clinical dosing thresholds. The exclusion of non‐English publications may have also introduced language bias.

## 5 Conclusions

BLT demonstrates antimicrobial activity against *S. aureus*, including MRSA, across experimental models. However, methodological variability, limited in vivo data, and uncertainty regarding the therapeutic window between antimicrobial effects and tissue safety limit clinical translation. Clarifying this therapeutic window through well‐designed studies is crucial for establishing BLT as a non‐antibiotic antimicrobial modality for *S. aureus*. Uniform adoption of the 8 proposed reporting parameters would enable meaningful meta‐analyses and support clinical translation.

## Funding

The authors have nothing to report.

## Conflicts of Interest

The authors declare no conflicts of interest.

## Supporting information


Supporting File 1

Supporting File 2

## Data Availability

The data that supports the findings of this study are available in the supporting information of this article.
