# Supplementary material for: The Use of Blue Light as an Antimicrobial Treatment Against Staphylococcus aureus: A Systematic Review
Source: Lasers Surg Med. 2026 Jul 15;58(9):715–25. doi: 10.1002/lsm.70172 (PMC13649102; doi:10.1002/lsm.70172)
Supplement: Supplementary file 1 [file LSM-58-715-s001.docx]

eFigure 1: PRISMA Flow Diagram

| **Study identification** | **Study type** | **Model type** | **Organism/strain** | **Wavelength (nm)** | **Dose (J/cm²)** | **Exposure time** | **Outcome summary** |
| --- | --- | --- | --- | --- | --- | --- | --- |
| ZHANG ET AL. (2023)^36^ | In Vivo | Rabbit Bone Implant | MRSA | 410;405 | Not reported | 10;20;30 min | 86.63±6.10%; 95.21±2.01%; 99.96±0.03% bactericidal rate * |
| ZHANG ET AL. (2023)^36^ | In Vitro | Planktonic | MRSA | 405 | 36 | 40 min | 5 log₁₀ reduction |
| NEGRI ET AL. (2025)^15^ | In Vivo | Porcine Wound Model | MRSA USA300 | 405 | 250 | 24-96 h post-infection | 1.5-4.7 log₁₀ reduction * |
| RUTALA ET AL. (2018)^40^ | In Vitro | Formica Sheet | MRSA (ATCC 43300) | 405±3 | Not reported | 0-72 h | 25-90% reduction |
| ONG ET AL. (2023)^40^ | Ex Vivo | Sheep skin + Ti implants | S. aureus ATCC 6538 biofilms | 405 | 135;270;540 | 15-60 min | No statistical difference vs pre-treatment |
| ONG ET AL. (2023)^13^ | Ex Vivo | Sheep skin + Ti implants | S. aureus ATCC 6538 biofilms | 405 | 1080;3240 | 6 h/day; 1 h twice daily (3 days) | 24h: 1.63 log₁₀ reduction; 72h: ~2.7 log₁₀ reduction |
| ESPADA ET AL. (2020)^38^ | In Vitro | MRSA Biofilm | MRSA (MEEI IB0004) | 405 | 216 | 60 min | 24h biofilm: 1.58 log₁₀ reduction; 48h biofilm: 0.20 log₁₀ reduction * |
| ESPADA ET AL. (2019)^9^ | In Vitro | Static Biofilm (24h) | MRSA USA300 | 405 | 216 | 60 min | 1.2 ± 0.20 log₁₀ reduction |
| ESPADA ET AL. (2019)^9^ | In Vitro | Static Biofilm (24h) | MRSA USA300 | 405 | 500 | 90 min | 3.48 ± 0.39 log₁₀ reduction |
| ESPADA ET AL. (2019)^9^ | In Vitro | Static Biofilm (48h) | MRSA USA300 | 405 | 216 | 60 min | 1.62 ± 0.13 log₁₀ reduction |
| ESPADA ET AL. (2019)^9^ | In Vitro | Static Biofilm (48h) | MRSA USA300 | 405 | 500 | 90 min | 2.35 ± 0.23 log₁₀ reduction |
| ESPADA ET AL. (2019)^9^ | In Vitro | Dynamic Biofilm | MRSA USA300 | 405 | 216 | 60 min | 2.05 ± 0.33 log₁₀ reduction ** |
| ESPADA ET AL. (2019)^9^ | In Vitro | Dynamic Biofilm | MRSA USA300 | 405 | 500 | 90 min | 2.58 ± 0.30 log₁₀ reduction ** |
| VOJTKOVSKA ET AL. (2025)^49^ | In Vitro | Agar Plate | S. aureus ATCC 25923 | 405 | Not reported | 8 h | ~0 log₁₀ reduction |
| VOJTKOVSKA ET AL. (2025)^49^ | In Vitro | Agar Plate | S. aureus ATCC 25923 | 405 | Not reported | 24 h | ~2 log₁₀ reduction (Trypticase only) |
| TOMB ET AL. (2017)^30^ | In Vitro | Bacterial Suspension | MSSA NCTC 4135 | 405 | 108 | 30 min | 1.2-1.3 log₁₀ reduction (15 cycles) ** |
| TOMB ET AL. (2017)^30^ | In Vitro | Bacterial Suspension | MRSA EMRSA-15 | 405 | 108 | 30 min | 1.3-1.4 log₁₀ reduction (15 cycles) ** |
| TOMB ET AL. (2017)^30^ | In Vitro | Bacterial Suspension | MSSA NCTC 4135 | 405 | 270 | 75 min | 4.8-5.2 log₁₀ reduction (15 cycles) ** |
| TOMB ET AL. (2017)^30^ | In Vitro | Bacterial Suspension | MRSA EMRSA-15 | 405 | 324 | 90 min | 5.3-5.4 log₁₀ reduction (15 cycles) ** |
| AL-SHAMMARY ET AL. (2020)^28^ | In Vitro | Liquid Culture | MRSA ATCC 43300 | 405 | Not reported | 1 min | 60% survival * |
| AL-SHAMMARY ET AL. (2020)^28^ | In Vitro | Liquid Culture | MRSA ATCC 43300 | 405 | Not reported | 2 min | 30% survival * |
| AL-SHAMMARY ET AL. (2020)^28^ | In Vitro | Liquid Culture | MRSA ATCC 43300 | 405 | Not reported | 5 min | 6% survival * |
| AL-SHAMMARY ET AL. (2020)^28^ | In Vitro | Liquid Culture | MRSA ATCC 43300 | 405 | 480 | 10 min | 6% survival* |
| MU ET AL. (2025)^34^ | In Vitro | Planktonic | S. aureus (DSM 799) | 453 | 3.45-165.6 | 2.5-120 min | Small decrease to decrease ** |
| MU ET AL. (2025)^34^ | In Vitro | Infected Implants | S. aureus (DSM 799) | 453 | 165.6 | 120 min (cycling) | No change ** |
| GALO ET AL. (2022)^50^ | In Vitro | Various | S. aureus ATCC 25923 | 470 | 157.78-284.90 | 3-6 h | 50.67-97.67% inhibition |
| NGUYEN ET AL. (2024)^46^ | In Vitro | Agar Plate | S. aureus ATCC 25923 | 405 | 11.51-184.13 | 5-80 min | 0-100% inactivation * |
| BIENER ET AL. (2017)^33^ | In Vitro | Bacterial Culture | MRSA USA300 | 405 | 121-242 | 15-30 min | ~3-10% survival |
| WU ET AL. (2018)^5^ | In Vitro | Bacterial Suspension | MRSA252 | 415 | 80 | Not reported | >6 log₁₀ reduction * |
| SHEHATOU ET AL. (2019)^47^ | In Vitro | Various | S. aureus strains | 405 | 62.7-540 | Various | 1.65-6.0 log₁₀ reduction * |
| ONG ET AL. (2023)^41^ | In Vitro | Planktonic Bacteria | S. aureus ATCC 6538 | 405 | Not reported | 2.5 h | 3.0 log₁₀ reduction * |
| ONG ET AL. (2023)^41^ | In Vitro | Planktonic/Biofilm | S. aureus ATCC 6538 | 405 | Not reported | 2.5-10 h | 0-3.0 log₁₀ reduction * |
| RAPACKA-ZDONCZYK ET AL. (2019)^22^ | In Vitro | Planktonic | S. aureus strains | 411 | 50-220 | 16-20h | 2-3 log₁₀ reduction * |
| LEANSE ET AL. (2020)^16^ | In Vitro/In Vivo | Various | USA300/AF0003 | 405;460 | 54-666 | 15-105 min | 0.28-3.58 log₁₀ reduction |
| GILLESPIE ET AL. (2017)^35^ | In Vitro | Liquid Suspension | S. aureus NCTC 4135 | 405 | 17.4-115 | 30-120 min | 48.9-99.8% reduction |
| AL-TAMEEMI ET AL. (2024)^51^ | In Vitro | Various surfaces | S. aureus USA300_LAC | 455 | 108 | 60 min | 25.8-50% survival |
| HOENES ET AL. (2021)^52^ | In Vitro | Bacterial Suspension | S. aureus (ATCC 43300) | 405;450 | 160-400 | Not reported | 1.22-5.69 log₁₀ reduction * |
| DOS ANJOS ET AL. (2020)^26^ | In Vitro | PBS/Milk | S. aureus ATCC 25923 | 413 | 720 | 120 min | >5 log₁₀ reduction |
| SINCLAIR ET AL. (2023)^48^ | In Vitro | Surface-seeded | S. aureus strains | 405 | 1.08-7.2 | 1-24 h | 15.2-100% reduction |
| HUI ET AL. (2025)^14^ | In Vitro/In Vivo | Various | MRSA USA300 | 410 | 54-738 | 3-41 h | Growth inhibition-2 log₁₀ reduction * |
| DOS ANJOS ET AL. (2023)^27^ | In Vitro | Bacterial Suspension | S. aureus ATCC 25923 | 410±10 | 63.5-549.6 | Not reported | 1.0-4.54 log₁₀ reduction * |
| MACLEAN ET AL. (2019)^31^ | In Vitro | Platelet Concentrates | S. aureus NCTC 4135 | 405 | 64.8-288 | 5-8 h | >92.5-99.6% reduction |
| BUMAH ET AL. (2015)^42^ | In Vitro | Agar plate | USA300 | 405;470 | 45-220 | Not reported | >3 log₁₀ reduction |
| BUMAH ET AL. (2020)^39^ | In Vitro | Planktonic/Biofilm | MRSA ATCC BAA-1680 | 450 | 4.5-10.8 | 1500-5400 s | 0.8-7.0 log₁₀ reduction |
| BUMAH ET AL. (2017)^43^ | In Vitro | Diamond Window | MRSA USA300 | 470 | 108-262 | 60-144 min | Complete suppression |
| BUMAH ET AL. (2015)^44^ | In Vitro | Agar plates | MRSA USA300 | 470 | 55 | 30.6 min | 90.2-97.3% suppression |
| MASSON-MEYERS ET AL. (2015)^45^ | In Vitro | Agar | MRSA USA300 | 405 | 40-121 | 15-240 min | 0-10% survival |
| YANG ET AL. (2024)^32^ | In Vitro | Planktonic | S. aureus ATCC25923 | 460 | 30-270 | 10-90 min | 0.64-4.11 log₁₀ reduction |
| HALSTEAD ET AL. (2019)^37^ | In Vitro | Biofilm | MSSA_10788 | 405-450 | 407.2-513 | 19.5-45 min | Variable biofilm effects ** |

eTable 1: Characteristics of Included Studies (n=33). In certain publications, multiple time points were evaluated. However, data was excluded from studies that did not provide results for the specified time points. Consequently, no time points were included in the table. If a time point was included but no results were specified, it was not included in the table. Outcome summaries labeled with a (*) indicates that the statistical significance was not specified. Outcome summaries denoted with (**) indicated that the time point was not statistically significant. Tables were organized by dose, with the highest dose listed first and subsequent rows displaying progressively lower doses.

| **Study identification** | **Study type** | **Model type** | **Organism/strain** | **Wavelength (nm)** | **Dose (J/cm²)** | **Exposure time** | **Outcome summary** | **Methodological quality assessment** |
| --- | --- | --- | --- | --- | --- | --- | --- | --- |
| LEANSE ET AL. (2020)^16^ | In Vitro/In Vivo | Various | MSSA | 405;460 | 54-666 | 15-105 min | 0.28-3.58 log₁₀ reduction | SYRCLE: unclear |
| HUI ET AL. (2025)^14^ | In Vitro/In Vivo | Various | MRSA | 410 | 54-738 | 3-41 h | Growth inhibition-2.0 log₁₀ reduction * | SYRCLE: high |
| ZHANG ET AL. (2023)^36^ | In Vivo | Rabbit Bone Implant | MRSA | 410;405 | Not reported | 10;20;30 min | 86.63±6.10%; 95.21±2.01%; 99.96±0.03% bactericidal rate * | SYRCLE: unclear |
| ONG ET AL. (2023)^13^ | Ex Vivo | Sheep skin + Ti implants | MSSA | 405 | 135;270;540 | 15-60 min | No statistical difference vs pre-treatment | Reported: wavelength, dose, exposure time; no dose verification |
| ONG ET AL. (2023)^13^ | Ex Vivo | Sheep skin + Ti implants | MSSA | 405 | 1080;3240 | 6 h/day; 1 h twice daily (3 days) | 24h: 1.63 log₁₀ reduction; 72h: ~2.7 log₁₀ reduction | Reported: wavelength, dose, exposure time; no dose verification |
| NEGRI ET AL. (2025)^15^ | In Vivo | Porcine Wound Model | MRSA | 405 | 250 | 24-96 h post-infection | 1.5-4.7 log₁₀ reduction | SYRCLE: unclear |

eTable 2. In Vivo and Ex Vivo Experiments (n=5). In certain publications, multiple time points were evaluated. However, data was excluded from studies that did not provide results for the specified time points. Consequently, no time points were included in the table. If a time point was included but no results were specified, it was not included in the table. Outcome summaries labeled with a (*) indicates that the statistical significance was not specified. Outcome summaries denoted with (**) indicated that the time point was not statistically significant. Tables were organized by dose, with the highest dose listed first and subsequent rows displaying progressively lower doses. Methodological quality assessment for in vivo studies was performed using the SYRCLE Risk of Bias tool and is summarized in the supplemental table.
